# Predicting the outcome of AIS brace treatment using expert judgement and a fuzzy model

**DOI:** 10.1186/1748-7161-10-S1-O63

**Published:** 2015-01-19

**Authors:** Eric Chalmers, Doug Hill, Vicky Zhao, Edmond Lou

**Affiliations:** 1 University of Alberta, Edmonton, AB T6G 2R3, Canada; 2 Alberta Health Services, Canada

## Objectives

This work measured how accurately the outcome of brace treatment can be predicted, using only information available at the start of treatment. The prediction accuracies of human experts and a fuzzy computer model were measured and compared.

## Methods

Data was obtained retrospectively from 28 AIS patients who had finished treatment (27 girls, 1 boy, aged 11-15 (mean 13), Cobb angles 20-44 degrees (mean 31), 21 daytime and 7 nighttime braces.) Patients were labelled 'progressed' if their Cobb angle had increased more than 5 degrees by the end of treatment, and 'non-progressed' otherwise.

A fuzzy model was developed to predict treatment outcome for each patient using clinical measurements taken at the first in-brace clinic. The model considers patient age, Cobb angle, Scoliometer measurement at the apex level, and in-brace Cobb angle correction. For each patient it calculated a probability-like score for each of three possible outcomes: 'progression' (Cobb angle increase > 5 degrees), 'neutral' (Cobb angle change of 0-5 degrees), and 'improvement' (Cobb angle decrease). For this study, the patient was predicted to progress if the 'progression' score was the highest.

Five AIS experts also participated: two orthopaedic surgeons, two orthotists, and one nurse practitioner. Participants were supplied with all available start-of-treatment clinical measurements for each patient, and asked to predict whether or not each patient would progress by the end of brace treatment. The multi-rater kappa was calculated to measure agreement between experts' predictions. The correlation of each expert's predictions and the model's predictions with the actual treatment outcome was measured.

## Results

Correlation between the fuzzy model's predictions and the actual outcomes was 0.7. Correlations between the five expert's predictions and the actual outcomes were 0.52, 0.52, 0.58, 0.46, and 0.71. The agreement among the human experts' predictions was k=0.43. Twelve of the twenty-eight patients had actually progressed; experts predicted 12-16 progressions, while the model predicted 17.

## Conclusions

Brace treatment outcome can be predicted at the start of treatment with moderate accuracy. The fuzzy model predicted treatment outcome more accurately than four human experts, and was comparable to the fifth. This type of fuzzy model works by estimating patient-specific likelihoods of several possible outcomes; future use of such models as decision-support aids may help inform clinical decision-making.

